# 4,4′,6,6′-Tetra­hydro­seleno-2,2′-[(*E*,*E*)-cyclo­hexane-1,2-diylbis(nitrilo­methyl­idyne)]diphenol

**DOI:** 10.1107/S1600536809031572

**Published:** 2009-08-15

**Authors:** Qiang Wang, Ru-Hua Zha, Ji-Wen Yuan, Qing-Fu Zeng

**Affiliations:** a Engineering Research Center for Clean Production of Textile Dyeing and Printing, Ministry of Education, Wuhan 430073, People’s Republic of China

## Abstract

In the title mol­ecule, C_20_H_22_N_2_O_2_Se_4_, the dihedral angle between the pendant aromatic rings is 67.1 (2)°. The conformation is stabilized by two intra­molecular O—H⋯N hydrogen bonds.

## Related literature

For background to the biological activity of Schiff base compounds, see: Shi *et al.* (2007 ▶). For reference structural data, see: Allen *et al.* (1987 ▶).
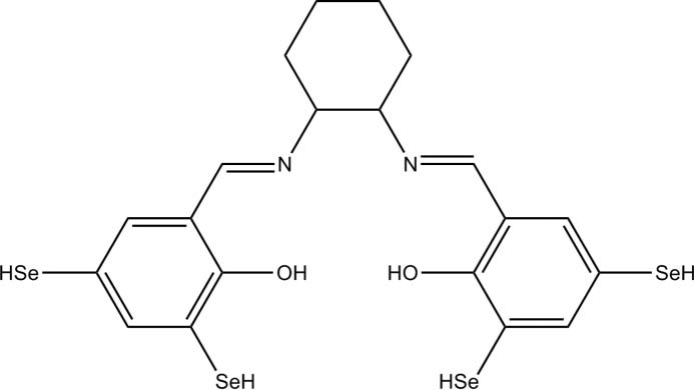


## Experimental

### 

#### Crystal data

C_20_H_22_N_2_O_2_Se_4_
*M* *_r_* = 638.24
Monoclinic, 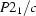

*a* = 15.493 (2) Å
*b* = 9.2975 (15) Å
*c* = 16.353 (2) Å
β = 109.865 (5)°
*V* = 2215.4 (5) Å^3^
*Z* = 4
Mo *K*α radiation
μ = 6.64 mm^−1^
*T* = 296 K
0.32 × 0.28 × 0.24 mm

#### Data collection

Enraf–Nonius CAD-4 diffractometer
Absorption correction: ψ scan (North *et al.*, 1968 ▶) *T*_min_ = 0.225, *T*_max_ = 0.299 (expected range = 0.153–0.203)
11901 measured reflections
4196 independent reflections
2743 reflections with *I* > 2σ(*I*)
*R*_int_ = 0.048
3 standard reflections every 200 reflections intensity decay: 1%

#### Refinement

*R*[*F* ^2^ > 2σ(*F* ^2^)] = 0.041
*wR*(*F* ^2^) = 0.105
*S* = 1.06
4196 reflections
255 parameters
H-atom parameters constrained
Δρ_max_ = 0.84 e Å^−3^
Δρ_min_ = −0.54 e Å^−3^

### 

Data collection: *CAD-4 Software* (Enraf–Nonius, 1989 ▶); cell refinement: *CAD-4 Software*; data reduction: *XCAD4* (Harms & Wocadlo, 1995 ▶); program(s) used to solve structure: *SHELXS97* (Sheldrick, 2008 ▶); program(s) used to refine structure: *SHELXL97* (Sheldrick, 2008 ▶); molecular graphics: *SHELXTL* (Sheldrick, 2008 ▶); software used to prepare material for publication: *SHELXTL*.

## Supplementary Material

Crystal structure: contains datablocks global, I. DOI: 10.1107/S1600536809031572/hb5041sup1.cif

Structure factors: contains datablocks I. DOI: 10.1107/S1600536809031572/hb5041Isup2.hkl

Additional supplementary materials:  crystallographic information; 3D view; checkCIF report

## Figures and Tables

**Table 1:** Hydrogen-bond geometry (Å, °)

*D*—H⋯*A*	*D*—H	H⋯*A*	*D*⋯*A*	*D*—H⋯*A*
O2—H2*A*⋯N2	0.82	1.85	2.575 (5)	147
O1—H1⋯N1	0.82	1.90	2.619 (5)	145

**Fig. 1.**
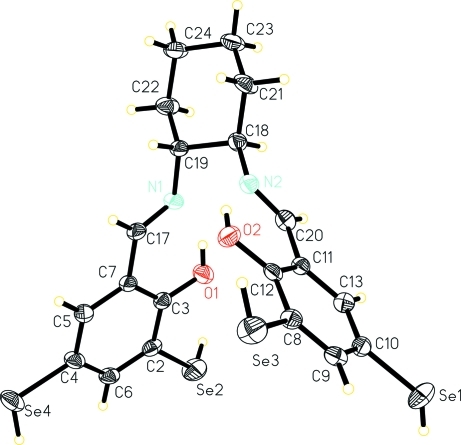
The molecular structure of (I) showing 30% probability displacement ellipsoids.

## supplementary crystallographic information

### Comment

There has been much research interest in Schiff base compounds due to their biological activities (Shi *et al.*, 2007). In this work, we report here the crystal structure of the title compound, (I). In (I), all bond lengths are within normal ranges (Allen *et al.*, 1987) (Fig. 1). There are two intramolecular O—H···N hydrogen bonds in (I) (Table 1).

### Experimental

A mixture of 3,5-dihydroseleno-2-hydroxybenzaldehyde (564 mg, 2 mmol) and cyclohexane-1,2-diamine (114 mg, 1 mmol) in methanol (10 ml) was stirred for 2 h. After keeping the filtrate in air for 5 d, yellow blocks of (I) were formed.

### Refinement

The Se bound H atoms were located in a difference map and refined as riding in their as-found relative positions. The C-bound H atoms were positioned geometrically (C—H = 0.93 Å for the aromatic H atoms and C—H = 0.96 Å for the aliphatic H atoms) and were refined as riding, with *U*_iso_(H) = 1.2*U*_eq_(C).

### Crystal data

**Table:**

C_20_H_22_N_2_O_2_Se_4_	*F*(000) = 1232
*M**_r_* = 638.24	*D*_x_ = 1.914 Mg m^−^^3^
Monoclinic, *P*2_1_/*c*	Mo *K*α radiation, λ = 0.71073 Å
Hall symbol: -P 2ybc	Cell parameters from 25 reflections
*a* = 15.493 (2) Å	θ = 9–12°
*b* = 9.2975 (15) Å	µ = 6.64 mm^−^^1^
*c* = 16.353 (2) Å	*T* = 296 K
β = 109.865 (5)°	Block, yellow
*V* = 2215.4 (5) Å^3^	0.32 × 0.28 × 0.24 mm
*Z* = 4	

### Data collection

**Table:**

Enraf–Nonius CAD-4 diffractometer	2743 reflections with *I* > 2σ(*I*)
Radiation source: fine-focus sealed tube	*R*_int_ = 0.048
graphite	θ_max_ = 25.7°, θ_min_ = 1.4°
ω/2θ scans	*h* = −18→18
Absorption correction: ψ scan (North *et al.*, 1968)	*k* = −9→11
*T*_min_ = 0.225, *T*_max_ = 0.299	*l* = −19→19
11901 measured reflections	3 standard reflections every 200 reflections
4196 independent reflections	intensity decay: 1%

### Refinement

**Table:**

Refinement on *F*^2^	Primary atom site location: structure-invariant direct methods
Least-squares matrix: full	Secondary atom site location: difference Fourier map
*R*[*F*^2^ > 2σ(*F*^2^)] = 0.041	Hydrogen site location: inferred from neighbouring sites
*wR*(*F*^2^) = 0.105	H-atom parameters constrained
*S* = 1.06	*w* = 1/[σ^2^(*F*_o_^2^) + (0.0494*P*)^2^] where *P* = (*F*_o_^2^ + 2*F*_c_^2^)/3
4196 reflections	(Δ/σ)_max_ = 0.001
255 parameters	Δρ_max_ = 0.84 e Å^−^^3^
0 restraints	Δρ_min_ = −0.54 e Å^−^^3^

### Special details

**Table:**

Geometry. All e.s.d.'s (except the e.s.d. in the dihedral angle between two l.s. planes) are estimated using the full covariance matrix. The cell e.s.d.'s are taken into account individually in the estimation of e.s.d.'s in distances, angles and torsion angles; correlations between e.s.d.'s in cell parameters are only used when they are defined by crystal symmetry. An approximate (isotropic) treatment of cell e.s.d.'s is used for estimating e.s.d.'s involving l.s. planes.
Refinement. Refinement of *F*^2^ against ALL reflections. The weighted *R*-factor *wR* and goodness of fit *S* are based on *F*^2^, conventional *R*-factors *R* are based on *F*, with *F* set to zero for negative *F*^2^. The threshold expression of *F*^2^ > σ(*F*^2^) is used only for calculating *R*-factors(gt) *etc*. and is not relevant to the choice of reflections for refinement. *R*-factors based on *F*^2^ are statistically about twice as large as those based on *F*, and *R*- factors based on ALL data will be even larger.

### Fractional atomic coordinates and isotropic or equivalent isotropic displacement parameters (Å^2^)

**Table:**

	*x*	*y*	*z*	*U*_iso_*/*U*_eq_	
Se1	1.08983 (4)	0.95743 (7)	1.27220 (3)	0.0629 (2)	
Se2	0.64176 (5)	0.45492 (8)	1.21754 (4)	0.0694 (2)	
Se3	0.87275 (4)	1.30994 (6)	1.00077 (4)	0.0618 (2)	
Se4	0.38089 (4)	0.88004 (8)	1.06267 (4)	0.0683 (2)	
O2	0.8152 (2)	1.0256 (4)	0.9129 (2)	0.0464 (9)	
H2A	0.8043	0.9482	0.8874	0.070*	
C20	0.8744 (3)	0.7422 (5)	0.9727 (3)	0.0397 (11)	
H20	0.8937	0.6520	0.9965	0.048*	
N2	0.8211 (2)	0.7512 (4)	0.8942 (2)	0.0400 (10)	
N1	0.6651 (3)	0.5930 (4)	0.8980 (2)	0.0414 (10)	
O1	0.6862 (2)	0.4896 (4)	1.0525 (2)	0.0544 (9)	
H1	0.6915	0.4905	1.0043	0.082*	
C8	0.9110 (3)	1.1261 (5)	1.0457 (3)	0.0373 (11)	
C2	0.5888 (3)	0.5808 (5)	1.1248 (3)	0.0414 (12)	
C17	0.6070 (3)	0.6798 (6)	0.9083 (3)	0.0412 (12)	
H17	0.5811	0.7480	0.8654	0.049*	
C9	0.9725 (3)	1.1108 (5)	1.1272 (3)	0.0399 (12)	
H9	0.9948	1.1914	1.1616	0.048*	
C10	1.0019 (3)	0.9748 (6)	1.1593 (3)	0.0388 (12)	
C11	0.9058 (3)	0.8696 (5)	1.0263 (3)	0.0341 (11)	
C3	0.6196 (3)	0.5804 (5)	1.0534 (3)	0.0384 (12)	
C18	0.7924 (3)	0.6202 (5)	0.8436 (3)	0.0383 (11)	
H18	0.8229	0.5374	0.8786	0.046*	
C4	0.4792 (3)	0.7638 (6)	1.0590 (3)	0.0441 (13)	
C5	0.5092 (3)	0.7665 (6)	0.9892 (3)	0.0448 (13)	
H5	0.4822	0.8297	0.9435	0.054*	
C6	0.5190 (3)	0.6701 (6)	1.1264 (3)	0.0431 (13)	
H6	0.4983	0.6672	1.1735	0.052*	
C7	0.5788 (3)	0.6770 (5)	0.9855 (3)	0.0384 (12)	
C12	0.8760 (3)	1.0062 (5)	0.9929 (3)	0.0343 (11)	
C19	0.6881 (3)	0.6039 (5)	0.8186 (3)	0.0381 (12)	
H19	0.6579	0.6888	0.7856	0.046*	
C13	0.9695 (3)	0.8555 (5)	1.1101 (3)	0.0391 (11)	
H13	0.9899	0.7648	1.1322	0.047*	
C21	0.8179 (3)	0.6275 (6)	0.7615 (3)	0.0564 (15)	
H21A	0.7892	0.7109	0.7275	0.068*	
H21B	0.8839	0.6378	0.7773	0.068*	
C22	0.6558 (4)	0.4706 (6)	0.7630 (3)	0.0545 (15)	
H22A	0.6815	0.3860	0.7974	0.065*	
H22B	0.5895	0.4642	0.7454	0.065*	
C23	0.7866 (3)	0.4919 (6)	0.7072 (4)	0.0566 (16)	
H23A	0.8183	0.4091	0.7398	0.068*	
H23B	0.8021	0.4990	0.6546	0.068*	
C24	0.6834 (3)	0.4719 (6)	0.6833 (3)	0.0553 (15)	
H24A	0.6517	0.5494	0.6451	0.066*	
H24B	0.6652	0.3819	0.6521	0.066*	
H2D	0.6487	0.3556	1.1959	0.066*	
H3D	0.8639	1.2980	0.9337	0.066*	
H1D	1.0891	0.9500	1.3302	0.066*	
H4D	0.3812	0.8789	1.1309	0.066*	

### Atomic displacement parameters (Å^2^)

**Table:**

	*U*^11^	*U*^22^	*U*^33^	*U*^12^	*U*^13^	*U*^23^
Se1	0.0686 (4)	0.0697 (5)	0.0371 (3)	−0.0124 (3)	0.0004 (3)	0.0052 (3)
Se2	0.0850 (5)	0.0807 (5)	0.0424 (3)	0.0074 (4)	0.0217 (3)	0.0183 (3)
Se3	0.0805 (4)	0.0374 (3)	0.0620 (4)	0.0104 (3)	0.0170 (3)	0.0004 (3)
Se4	0.0677 (4)	0.0887 (5)	0.0628 (4)	0.0210 (4)	0.0410 (3)	−0.0011 (3)
O2	0.049 (2)	0.046 (2)	0.0379 (19)	0.0044 (19)	0.0062 (17)	−0.0063 (16)
C20	0.043 (3)	0.031 (3)	0.048 (3)	0.002 (2)	0.019 (3)	0.002 (2)
N2	0.035 (2)	0.045 (3)	0.042 (2)	−0.003 (2)	0.0160 (19)	−0.005 (2)
N1	0.042 (2)	0.054 (3)	0.034 (2)	−0.001 (2)	0.0202 (19)	−0.003 (2)
O1	0.055 (2)	0.067 (3)	0.046 (2)	0.014 (2)	0.0226 (19)	0.0060 (19)
C8	0.042 (3)	0.037 (3)	0.036 (3)	0.000 (2)	0.017 (2)	0.002 (2)
C2	0.043 (3)	0.052 (3)	0.028 (2)	−0.012 (3)	0.011 (2)	−0.001 (2)
C17	0.040 (3)	0.057 (4)	0.028 (2)	−0.002 (3)	0.013 (2)	0.001 (2)
C9	0.046 (3)	0.035 (3)	0.047 (3)	−0.005 (3)	0.026 (3)	−0.006 (2)
C10	0.034 (3)	0.052 (3)	0.033 (3)	−0.003 (3)	0.013 (2)	0.001 (2)
C11	0.032 (2)	0.038 (3)	0.035 (3)	−0.003 (2)	0.014 (2)	−0.005 (2)
C3	0.034 (3)	0.048 (3)	0.033 (3)	−0.008 (2)	0.011 (2)	−0.006 (2)
C18	0.036 (3)	0.037 (3)	0.043 (3)	−0.002 (2)	0.015 (2)	−0.009 (2)
C4	0.044 (3)	0.055 (3)	0.042 (3)	−0.003 (3)	0.025 (2)	−0.005 (3)
C5	0.043 (3)	0.060 (4)	0.034 (3)	0.005 (3)	0.016 (2)	0.003 (2)
C6	0.045 (3)	0.057 (4)	0.033 (3)	−0.011 (3)	0.021 (2)	−0.006 (3)
C7	0.033 (3)	0.054 (3)	0.030 (2)	−0.003 (2)	0.013 (2)	−0.005 (2)
C12	0.033 (3)	0.040 (3)	0.036 (3)	0.005 (2)	0.019 (2)	−0.001 (2)
C19	0.036 (3)	0.052 (3)	0.030 (2)	−0.005 (2)	0.015 (2)	−0.004 (2)
C13	0.043 (3)	0.037 (3)	0.039 (3)	0.000 (3)	0.015 (2)	0.003 (2)
C21	0.047 (3)	0.073 (4)	0.065 (3)	−0.020 (3)	0.040 (3)	−0.023 (3)
C22	0.052 (3)	0.071 (4)	0.049 (3)	−0.023 (3)	0.029 (3)	−0.018 (3)
C23	0.056 (3)	0.070 (4)	0.055 (3)	−0.012 (3)	0.034 (3)	−0.027 (3)
C24	0.057 (3)	0.074 (4)	0.043 (3)	−0.018 (3)	0.028 (3)	−0.019 (3)

### Geometric parameters (Å, °)

**Table:**

Se1—C10	1.893 (5)	C10—C13	1.362 (7)
Se1—H1D	0.9542	C11—C13	1.396 (6)
Se2—C2	1.869 (5)	C11—C12	1.398 (6)
Se2—H2D	1.0078	C3—C7	1.400 (6)
Se3—C8	1.875 (5)	C18—C21	1.524 (6)
Se3—H3D	1.0645	C18—C19	1.534 (6)
Se4—C4	1.885 (5)	C18—H18	0.9800
Se4—H4D	1.1136	C4—C5	1.372 (6)
O2—C12	1.340 (5)	C4—C6	1.376 (7)
O2—H2A	0.8200	C5—C7	1.380 (6)
C20—N2	1.271 (6)	C5—H5	0.9300
C20—C11	1.455 (7)	C6—H6	0.9300
C20—H20	0.9300	C19—C22	1.518 (7)
N2—C18	1.455 (6)	C19—H19	0.9800
N1—C17	1.262 (6)	C13—H13	0.9300
N1—C19	1.461 (5)	C21—C23	1.523 (7)
O1—C3	1.337 (6)	C21—H21A	0.9700
O1—H1	0.8200	C21—H21B	0.9700
C8—C9	1.357 (6)	C22—C24	1.503 (6)
C8—C12	1.402 (6)	C22—H22A	0.9700
C2—C6	1.371 (6)	C22—H22B	0.9700
C2—C3	1.404 (6)	C23—C24	1.524 (7)
C17—C7	1.470 (6)	C23—H23A	0.9700
C17—H17	0.9300	C23—H23B	0.9700
C9—C10	1.385 (7)	C24—H24A	0.9700
C9—H9	0.9300	C24—H24B	0.9700
			
C10—Se1—H1D	136.7	C7—C5—H5	119.4
C2—Se2—H2D	111.0	C2—C6—C4	120.7 (4)
C8—Se3—H3D	102.8	C2—C6—H6	119.7
C4—Se4—H4D	107.3	C4—C6—H6	119.7
C12—O2—H2A	109.5	C5—C7—C3	120.1 (4)
N2—C20—C11	121.6 (5)	C5—C7—C17	119.4 (4)
N2—C20—H20	119.2	C3—C7—C17	120.4 (4)
C11—C20—H20	119.2	O2—C12—C11	122.2 (4)
C20—N2—C18	119.2 (4)	O2—C12—C8	119.4 (4)
C17—N1—C19	118.3 (4)	C11—C12—C8	118.3 (4)
C3—O1—H1	109.5	N1—C19—C22	110.2 (4)
C9—C8—C12	121.1 (5)	N1—C19—C18	108.9 (4)
C9—C8—Se3	120.3 (4)	C22—C19—C18	110.1 (4)
C12—C8—Se3	118.6 (3)	N1—C19—H19	109.2
C6—C2—C3	120.7 (4)	C22—C19—H19	109.2
C6—C2—Se2	119.7 (3)	C18—C19—H19	109.2
C3—C2—Se2	119.6 (4)	C10—C13—C11	120.0 (5)
N1—C17—C7	122.5 (4)	C10—C13—H13	120.0
N1—C17—H17	118.8	C11—C13—H13	120.0
C7—C17—H17	118.8	C23—C21—C18	110.5 (4)
C8—C9—C10	119.9 (5)	C23—C21—H21A	109.5
C8—C9—H9	120.0	C18—C21—H21A	109.5
C10—C9—H9	120.0	C23—C21—H21B	109.5
C13—C10—C9	120.8 (4)	C18—C21—H21B	109.5
C13—C10—Se1	120.4 (4)	H21A—C21—H21B	108.1
C9—C10—Se1	118.8 (4)	C24—C22—C19	112.3 (4)
C13—C11—C12	119.9 (4)	C24—C22—H22A	109.1
C13—C11—C20	119.7 (5)	C19—C22—H22A	109.1
C12—C11—C20	120.4 (4)	C24—C22—H22B	109.1
O1—C3—C7	122.6 (4)	C19—C22—H22B	109.1
O1—C3—C2	119.4 (4)	H22A—C22—H22B	107.9
C7—C3—C2	118.0 (4)	C21—C23—C24	110.4 (4)
N2—C18—C21	110.1 (4)	C21—C23—H23A	109.6
N2—C18—C19	108.9 (4)	C24—C23—H23A	109.6
C21—C18—C19	109.6 (4)	C21—C23—H23B	109.6
N2—C18—H18	109.4	C24—C23—H23B	109.6
C21—C18—H18	109.4	H23A—C23—H23B	108.1
C19—C18—H18	109.4	C22—C24—C23	111.2 (4)
C5—C4—C6	119.5 (4)	C22—C24—H24A	109.4
C5—C4—Se4	121.5 (4)	C23—C24—H24A	109.4
C6—C4—Se4	119.0 (3)	C22—C24—H24B	109.4
C4—C5—C7	121.1 (5)	C23—C24—H24B	109.4
C4—C5—H5	119.4	H24A—C24—H24B	108.0

### Hydrogen-bond geometry (Å, °)

**Table:**

*D*—H···*A*	*D*—H	H···*A*	*D*···*A*	*D*—H···*A*
O2—H2A···N2	0.82	1.85	2.575 (5)	147
O1—H1···N1	0.82	1.90	2.619 (5)	145

## References

[bb1] Allen, F. H., Kennard, O., Watson, D. G., Brammer, L., Orpen, A. G. & Taylor, R. (1987). *J. Chem. Soc. Perkin Trans. 2*, pp. S1–19.

[bb2] Enraf–Nonius (1989). *CAD-4 Software* Enraf–Nonius, Delft, The Netherlands.

[bb3] Harms, K. & Wocadlo, S. (1995). *XCAD4* University of Marburg, Germany.

[bb4] North, A. C. T., Phillips, D. C. & Mathews, F. S. (1968). *Acta Cryst.* A**24**, 351–359.

[bb5] Sheldrick, G. M. (2008). *Acta Cryst.* A**64**, 112–122. 10.1107/S0108767307043930 18156677

[bb6] Shi, L., Ge, H.-M., Tan, S.-H., Li, H.-Q., Song, Y.-C., Zhu, H.-L. & Tan, R.-X. (2007). *Eur. J. Med. Chem.* **42**, 558–564. 10.1016/j.ejmech.2006.11.010 17194508

